# General Anesthesia Without Nerve Block Is Non-Inferior to General Anesthesia with Nerve Block for Postoperative Pain Control in Antegrade Femoral Limb Lengthening: A Retrospective Study †

**DOI:** 10.3390/jcm14124066

**Published:** 2025-06-09

**Authors:** Akram Al Ramlawi, Zhongming Chen, Michael Assayag, John E. Herzenberg, Philip K. McClure

**Affiliations:** International Center for Limb Lengthening, Sinai Hospital of Baltimore, Baltimore, MD 21215, USA; zchen2@lifebridgehealth.org (Z.C.); massayag@lifebridgehealth.org (M.A.); jherzenb@lifebridgehealth.org (J.E.H.); pmcclure@lifebridgehealth.org (P.K.M.)

**Keywords:** limb lengthening, lower limb, anesthetic block, femoral nerve, fascia iliaca, intramedullary lengthening nail, pain management, pain control, non inferiority, general anesthesia

## Abstract

**Background**: Effective postoperative pain management is essential in femoral limb lengthening surgery. Although regional nerve blocks reduce pain and opioid use, their benefit in antegrade femoral intramedullary limb lengthening is unclear. This study compares postoperative pain and opioid consumption in patients receiving general anesthesia (GA) alone versus GA with a preoperative femoral or fascia iliaca nerve block. **Methods**: A retrospective review included 192 patients who underwent femoral lengthening with intramedullary telescoping nails between January 2012 and October 2023 at a single center. Patients were categorized into Group A (GA alone, *n* = 131) and Group B (GA plus nerve block, *n* = 61). Primary outcomes were postoperative mean and maximum pain scores in the first 24 h, total opioid pills prescribed at discharge, and total morphine milligram equivalents (MMEs) used in the Post-Anesthesia Care Unit (PACU). Non-inferiority was defined by a margin of one standard deviation for pain scores and opioid usage. **Results**: Demographics were similar between groups. Maximum PACU pain scores were 3.8 for Group A and 3.3 for Group B (*p* > 0.05); mean pain scores were 2.1 and 1.9, respectively (*p* > 0.05). GA alone was non-inferior for pain control. However, total opioid pills prescribed at discharge were higher in Group A (23.2) than Group B (10) (*p* < 0.05). PACU MME usage was also higher in Group A (26 vs. 18.4 ± 15 mg, *p* < 0.05), though non-inferiority criteria were met. **Conclusions**: GA alone is non-inferior to GA with nerve block for postoperative pain management following antegrade femoral intramedullary limb lengthening. Although patients without a nerve block received more opioids at discharge, their pain control remained similarly effective. Given potential risks and the lack of clear pain reduction benefits, routine nerve block use may not be warranted. Decisions regarding nerve block application should be individualized, considering patient preferences, surgeon recommendations, and anesthesiologist input.

## 1. Introduction

Pain management post-limb lengthening can be a challenge for both the care team and the patient, with some patients requiring an overnight stay. Typically, intramedullary limb lengthening is performed under general anesthesia (GA) and postoperative pain is managed with opioid or opioid derivatives. The side effects associated with narcotics such as nausea and drowsiness along with the cost associated with a prolonged hospital stay encourage medical professionals taking care of patients to find more efficient means to decrease operative-related pain. At our institution, an increasing number of patients are opting for outpatient surgery, which raises the need for predictable pain control with minimal complications. We hypothesize that oral or intravenous analgesia is non-inferior to nerve block for postoperative pain control.

Regional nerve block use has been hypothesized to decrease the time to discharge and postoperative narcotic consumption after both elective and emergent operative procedures [1,2]. Whether for shoulder [1,2], hip [3,4], knee [5], or ankle [6,7,8] surgeries, regional nerve blocks have been shown to be effective in decreasing hospital stays and postoperative opioid use. Similar results have been published for pediatric femur trauma [9].

In addition to providing sensory innervation to the anterior thigh regions, the femoral nerve also supplies sensation to the quadriceps femoris muscle [10,11]. While it has been suggested that femoral nerve blocks offer substantial analgesic benefits and reduce the need for opioids, they can also lead to motor blockade of the quadriceps, potentially resulting in delayed functional mobilization and an elevated risk of falls.

Recent studies have also shown that patients may experience increased rebound pain after nerve block effect fades away during the first 24 to 48 h postoperatively [8]. A retrospective study looking at patients receiving a single-injection femoral nerve block for ACL (anterior cruciate ligament) repair found that on average, 33 h of continuous nerve blockade was needed to lower rebound pain by 1 point over a 10-point scale [12]. Another retrospective analysis identified severe rebound pain in around half of the ambulatory surgery patients who received a regional block, with bone surgery or injury being one of the major risk factors for rebound pain [13].

Moreover, nerve identification and anesthetic injection is a time-consuming procedure in the setting of the operation room, with each minute costing around USD 16.27 without factoring in the professional fees of anesthesiologists and surgeons or the costs of medications, instruments, and implants [14].

In light of these findings, and due to the lack of studies investigating nerve block use among antegrade femoral intramedullary limb lengthening surgeries, the aim of this study is to examine the difference in postoperative pain and opioid intake among patients undergoing limb lengthening surgery using antegrade intramedullary nails with or without a femoral and/or fascia iliaca nerve block. We hypothesize a non-inferiority in outcomes stated previously for patients undergoing GA with and without a regional single nerve block.

## 2. Methods

This is an institutional review board (IRB)-approved retrospective review of patients who underwent femoral lengthening surgery using intramedullary telescoping nails at a single orthopedic center during the period between January 2012 and October 2023. Patient consent was waived by the hospital’s IRB (45 CFR 46.104(d)(4)). Patients with a known history of morphine dependance were excluded from the study. Patients undergoing concurrent procedures or procedures within a month of the index procedure, other than femoral lengthening using an antegrade intramedullary nail, and patients not presenting for 1 or more follow-up visits, were also excluded from the study. Patient collection was performed using non-probabilistic convenience sampling. Patients underwent limb lengthening procedures primarily due to congenital conditions (40%), cosmetic height gain (35%), and traumatic injuries (25%). These indications were similarly distributed between Group A (general anesthesia alone) and Group B (general anesthesia plus nerve block), with no statistically significant difference observed between the groups (*p* > 0.05).

We assigned a non-inferiority margin of 1 SD (on a 10-point numerical rating scale) difference in mean pain and maximum reported pain during hospital stay and total opioid medication prescription pills at home. The choice of a non-inferiority margin set at one standard deviation (SD) was based on established standards in clinical pain research, particularly considering the minimal clinically important difference (MCID) reported in the literature. Previous studies have indicated that clinically meaningful changes in pain intensity typically require at least a 1.65-point shift on a 10-point pain scale. Given the relatively low baseline pain scores observed in antegrade femoral limb lengthening procedures, adopting a narrower margin might detect statistically significant but clinically insignificant differences. Conversely, a wider margin could mask clinically relevant differences. Thus, selecting one SD as the non-inferiority threshold provides a clinically meaningful and methodologically sound balance [15]. A treatment was considered non-inferior if the lower bound of the 95% confidence interval (CI) does not intersect the inferiority line. In case that the 95% CI intersected with the non-inferiority margin, the comparison was deemed as inconclusive.

We defined the primary outcomes as both postoperative mean pain scores and maximum pain scores during the first 24 h of hospital stay, number of opioid pills upon home discharge and during subsequent related visits, and total morphine milligram equivalents (MMEs) in the Post-Anesthesia Care Unit (PACU). We also compared femoral nerve blocks with fascia iliaca nerve blocks as our secondary outcome. The decision on whether or not to perform a nerve block was made by the patient, anesthesiologist, and surgeon.

Patients receiving only general anesthesia (GA) were assigned to Group A, while patients receiving a preoperative nerve block with or without another nerve block before the induction of GA were assigned into Group B. Patients in both groups were matched by age and gender. Both groups were then compared. A staff anesthesiologist performed all nerve blocks under ultrasound guidance. For the femoral nerve block, the needle tip was successfully positioned near the nerve under ultrasound guidance, and 15 to 25 mL of ropivacaine (0.33–0.75%) was administered in 5 mL increments to ensure circumferential distribution around the femoral nerve. For fascia iliaca nerve blocks, 3 mL/kg (up to a maximum of 40 mL) of ropivacaine 0.5% was injected between the iliac fascia and the iliopsoas muscle, also using ultrasound guidance. Patients were transferred to the PACU and subsequently either to the Surgical Day Care Unit (SDCU) or discharged home. Patients were allowed to go home when they fulfilled the institutional discharge criteria for ambulatory surgery, with the majority having an overnight observation by default as per surgeon’s preference.

Age, sex, height, weight, body mass index (BMI), and operative time among other demographic data were collected. For categorical variables, groups A and B were compared via chi-square analyses. Independent-sample Student *t*-tests were used for continuous variables. A *p* value < 0.05 was considered to be statistically significant. Data analyses and collection were conducted as per the hospital’s institutional ethical review board. The normality of continuous data was assessed using the Shapiro–Wilk test prior to selecting the statistical tests. All continuous variables demonstrated normal distribution, thus justifying the use of independent-sample Student’s *t*-tests for comparisons between groups.

Patient-controlled analgesia pumps (PCA) with morphine were used in some of the patients undergoing limb lengthening before 22 June 2017.

## 3. Results

A total of 230 consecutive antegrade femoral intramedullary limb lengthening surgeries were performed between 30 March 2012 and 31 October 2023, of whom 192 met the inclusion criteria. In total, 15 patients were excluded for undergoing concomitant tibial osteotomies, 10 for known previous morphine use, 10 for lost follow-up data, and 3 for deficiencies in records. Of these patients, 131 had standard GA without a nerve block (Group A) via a combination of anesthetic agents, and 61 patients had GA in addition to a preoperative nerve block (Group B). Patients were grouped depending on whether nerve blockade was applied in addition to general anesthesia.

The GA group without a nerve block (Group A) consisted of 77 male and 54 female patients, with an average age of 20.1 ± 12.85 years. The mean BMI was 25.99 kg/m^2^. Thirty-one patients were equipped with PCA pumps. Twenty patients underwent bilateral limb lengthening.

The indications for limb lengthening surgery were evenly distributed between groups: congenital cases comprised approximately 40% of the cohort, cosmetic cases accounted for about 35%, and traumatic cases represented the remaining 25%. No significant differences were identified between Group A and Group B regarding these distributions (*p* = 0.65).

Patients undergoing at least a femoral nerve block without a fascia iliaca block (33) and those with a femoral nerve block with a fascia iliaca block (28) (Group B) consisted of 30 male and 31 female patients, with a mean age of 21.39 ± 14.26 years. The mean BMI was 25.42 kg/m^2^. Nine patients were equipped with PCA pumps. Twelve patients underwent bilateral limb lengthening.

No statistically significant difference was appreciated among the two groups with regard to sex (*p* = 0.72), age (*p* = 0.84), BMI (*p* = 0.87), PCA (*p* = 0.79), or bilateral lengthening (*p* = 0.88) (Table 1).

Patients’ reported max pain score on a scale from 0 to 10 during PACU admission was 3.8 ± 2.3 for Group A and 3.3 ± 2.5 for Group B. Similar trends were seen for average pain during admission, with an average mean pain of 2.1 ± 1.7 for Group A and 1.9 ± 2 for Group B. The difference between groups was not statistically significant (*p* = 0.71).

**Figure 1:**
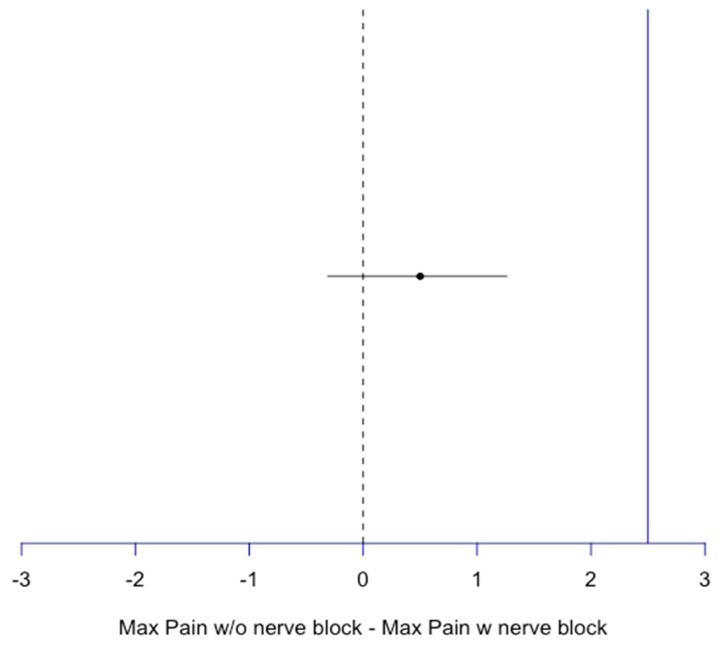
Non-inferiority plot of maximum pain for block compared to no block patients. Difference = 0.5, CI [−0.31, 1.26], blue line: non-inferiority line = 1 SD of max pain w nerve block = 2.5.

**Figure 2:**
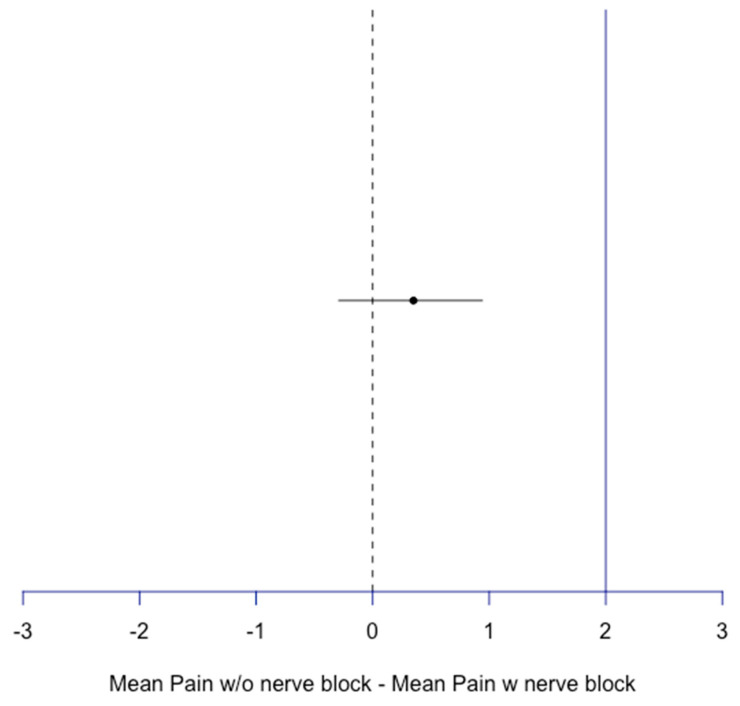
Non-inferiority plot of mean pain for block compared to no block patients. Difference = 0.35, CI [−0.29, 0.94], blue line: non-inferiority line = 1 SD of max pain w nerve block = 2.

As the non-inferiority limit was considered one standard deviation away from the maximum and average pain reported by Group B, treatment without a nerve block was deemed non-inferior to nerve block treatment since its confidence ratio does not overlap with the non-inferiority line (CI = −0.31, 1.26 for maximum average pain difference and −0.29, 0.94 for mean average pain difference) (Figure 1 and Figure 2).

**Figure 3:**
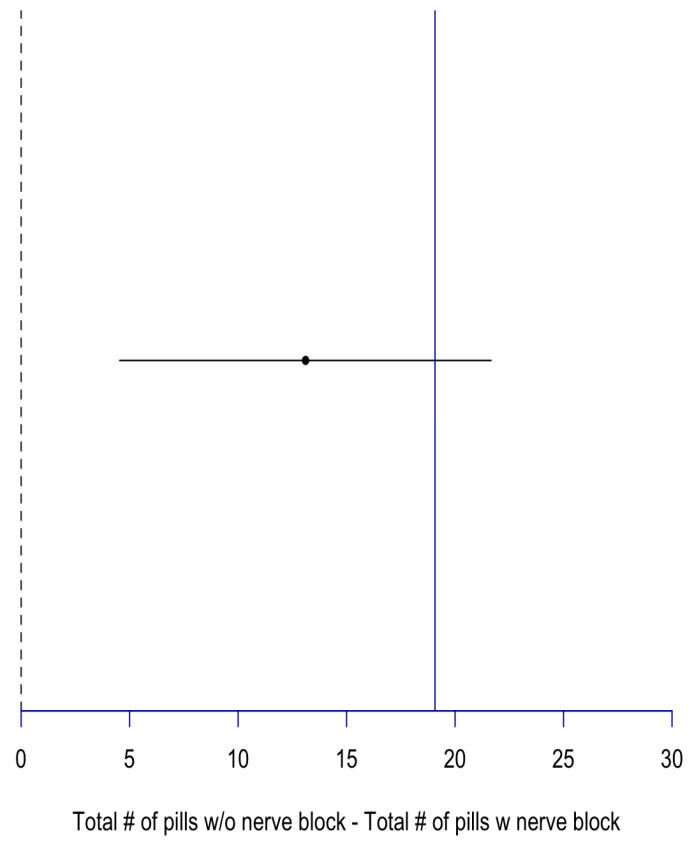
Non-inferiority plot of total number of pills prescribed for block compared to no block patients. Total number of pills difference = 13.11, CI [4.54, 21.66]; blue line: non-inferiority line = 1 SD of total number of pills w nerve block = 19.07.

On average, the total number of pills for Group A was 23.2 ± 35.1, and 10 ± 19 for Group B. The difference between both groups was statistically significant (*p* = 0.56). With non-inferiority lines deemed to be 1 SD (19) away from the block group mean, our results show the inferiority of no block usage compared to block usage in terms of total number of pills on discharge (CI = 4.54, 21.66). Furthermore, 12% (23 patients) of our study cohort requested a narcotic drug refill, with 11.5% (15 patients) of those from Group A and 13.3% (8 patients) from Group B (*p* = 0.41) (Figure 3).

**Figure 4:**
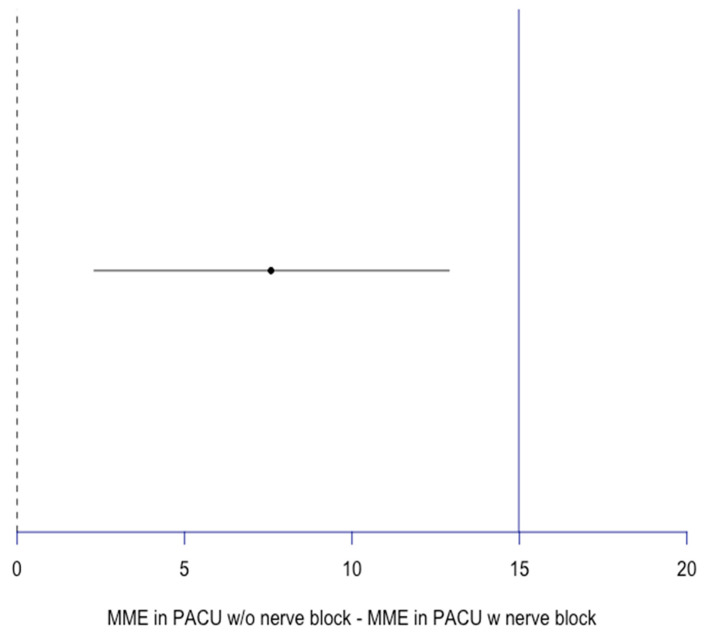
Non-inferiority plot for MME in PACU W/O nerve block compared to MME in PACU W nerve block. MME in PACU difference = 7.6 mg, CI [2.3, 12.9]; blue line: non-inferiority line = 1 SD of MME in PACU w nerve block = 14.98. MME: morphine milligram equivalent. PACU: Post Anesthesia Care Unit.

Furthermore, the MME taken in the PACU was 23.6 ± 19.6 mg overall: 26 ± 21 mg for Group A and 18.4 ± 15 mg for Group B (*p* < 0.05). With non-inferiority lines deemed to be 1 SD (15) away from the block group mean, our results show the non-inferiority of no block usage compared to block usage in terms of PACU MME on discharge with a confidence interval of 2.3, 12.9 (Figure 4).

PCA pumps with morphine were used in 17 cases. Seventeen patients in Group A used a PCA pump, which accounts for 16% of the group that did not receive a nerve block(s). Ten patients in Group B used a PCA pump, which accounts for 17% of the group that did receive nerve blocks. The difference between both groups was not statistically significant (*p* = 0.16).

In comparing pain management strategies involving a femoral nerve block alone versus a femoral nerve block combined with a fascia iliaca block, no statistically significant difference was observed in mean pain scores (*p* = 0.28), with patients in the combined block group reporting a lower mean pain score of 1 compared to a score of 2 in the femoral nerve block group. Similarly, no significant differences were found in maximum pain scores, total number of pills administered, or MME received between the two groups (*p* = 0.66). Specifically, while the maximum pain score was slightly lower in the combined block group (2.3) compared to the femoral nerve block group (3.3), the differences were not statistically significant. Similarly, there were no significant differences in the total number of pills administered (13 in the combined block group vs. 7 in the femoral nerve block group) or MME (19 in the combined block group vs. 25 in the femoral nerve block group) (Table 2).

There was no statistical difference between patients with PCA pumps and those without PCA pumps in terms of the number of pills prescribed upon discharge (*p* = 0.43), max pain (*p* = 0.24), or mean pain (*p* = 0.18).

A post hoc power analysis confirmed our sample size provided over 80% power to detect differences at the chosen non-inferiority margin.

## 4. Discussion

Our study compared antegrade intramedullary femoral limb lengthening with and without a regional intraoperative single nerve block. We hypothesized a non-inferiority relationship between general anesthesia alone compared to using general anesthesia with an intra-op femoral nerve block with and without a fascia iliaca nerve block for intramedullary femoral lengthening procedures. We retrospectively examined 192 surgeries and found both the nerve block and no nerve block groups to be demographically similar. We also found a similar distribution of PCA pump use among both groups, and no significant difference between PCA pump use for maximum and mean pain or total number of pills prescribed after discharge. Furthermore, we investigated maximum and mean pain during hospital stay after surgery and the total number of opioid pills prescribed at discharge and during subsequent visits. Finally, we examined PACU MME intake, which was found to be more in the study cohort that did not receive nerve block. However, we found that general anesthesia without a nerve block was non-inferior to general anesthesia with a nerve block in all the mentioned variables except for the total number of pills prescribed after discharge. In terms of secondary outcomes, we found a femoral block to be equivalent to femoral and fascia iliaca blocks in terms of maximum reported pain, number of pills, and MME, and average pain during the first 24 h of admission.

Our primary outcome was average and maximum postoperative pain as a significant patient-centered morbidity [13,16]. Our results run seemingly counter to previously published studies in different orthopedic fields [9,17,18], as we found no significant decrease in patients’ pain during their hospital admission regardless of nerve block application or lack thereof. Although previous work has shown superiority of a single-dose nerve block in trauma or arthroplasty procedures, these cases are typically present with or require extensive soft tissue insult, whereas the percutaneous osteotomy (at the proximal 1/3rd of the femoral shaft) in intramedullary limb lengthening happens in a controlled manner with minimal soft tissue injury. This difference might explain the benefit or lack thereof of a single-dose nerve block. Furthermore, Ward et al. [19] explored nerve blocks for early pain management in hip arthroscopy procedures and found a significant increase in patient satisfaction compared to patients taking morphine for analgesia. Although Ward et al. [19] reported increased satisfaction with nerve blocks, they did not report overall pain scores during admission. Patient satisfaction is a subjective measure that may not accurately reflect pain relief. Nonetheless, in accordance with our findings, Rowlands et al. [17], in a randomized controlled clinical trial, examined nerve blocks for hip fractures and found no benefit of block application for dynamic pain relief nor a decrease in postoperative pain. Although our study included patient-reported pain, it is unclear if reported pain was static or dynamic. Most likely, patients report an average of both. Whereas Huh et al. reported statistically significant pain relief in patients undergoing single-injection nerve blocks in hip fracture patients, the difference was not clinically significant, with a Visual Analog Scale (VAS) score difference of 1.7 at hour 6 postoperatively, 0.37 at hour 12 postoperatively, and no statistically significant difference after hour 12. Furthermore, the study did not address pain difference during the first 6 h post-op [20].

Although patients undergoing a nerve block required less morphine or morphine equivalent, on average, the difference was not deemed clinically significant, and no block was deemed non-inferior to block application in terms of MME use. Our results are further strengthened by a study by Dold et al. examining the benefit of a preoperative nerve block versus no block, as that study reported no statistically significant decrease in the use of oxycodone in PACU in patients belonging to the femoral block group [3]. A later randomized controlled trial that investigated a preoperative nerve block versus saline injection for hip arthroscopy found no benefit of a nerve block in terms of opioid consumption during hospitalization for patients, along with a considerable increase in the risk of fall due to muscle weakness [21]. The study concluded that the routine use of a nerve block for outpatient hip arthroscopy cannot be recommended. In a systematic review, Kim et al. found no benefit of a nerve block over local anesthesia for hip arthroscopy. The authors attributed their findings to the minimally invasive nature of hip arthroscopy, which typically involves less extensive anatomical alterations compared to open hip surgeries like hip arthroplasty. They also highlighted the preservation of extracapsular structures as a contributing factor. Consequently, the authors concluded that the impact of a peripheral nerve block may be insignificant in such cases. We posit that a similar rationale applies to antegrade limb lengthening procedures. Essentially, both arthroscopy and antegrade lengthening are considered relatively minor interventions, thus potentially rendering a nerve block unnecessary [22].

The previous literature has hypothesized the existence of rebound pain 24 to 48 h proceeding preoperative nerve block application, due to the reduced amount of anesthetic used during surgery [12,13]. Although our study did not elucidate the exact timing of maximum postoperative pain, we believe that it should be correlated to the timing of the rebound pain described previously. Further research is needed to explore the relationship between rebound pain and nerve blocks in limb lengthening patients. With the increase in same-day limb lengthening surgeries, rebound pain might hold considerable implications since patients will experience excruciating pain once they are at home and without appropriate medical support for adequate pain relief.

In addition, the goal of pain management after surgery is to manage the pain but not completely remove it, as some degree of postoperative pain is important for patients to understand their physical limitations and avoid activities that might hinder their recovery. On average, pain ratings of 4 or less (mild-to-moderate pain intensity) have been well-tolerated by patients who have undergone surgery [23,24]. The average postoperative pain score in our non-block group was 2.1, significantly below the proposed threshold of 4, indicating a lack of need for pain intervention.

Moreover, Bahreini et al. [15] studied the minimal clinically important difference (MCID), which is the minimal change in VAS that is correlated to meaningful pain change. The study found that the VAS needs to be changed by a minimum of 1.65, on average, for patients to report any meaningful change in pattern. Our study found an average difference between the block and no block groups to be 0.35 for average pain during PACU admission and 0.5 for max pain, which is considerably less than the proposed MCID of 1.65.

In terms of the number of opioid pills prescribed after discharge and during subsequent visits, we found no block application to be non-inferior to block application, reflecting similar outcomes in chronic pain relief among both groups. Furthermore, the number of pills prescribed could exaggerate the number of pills taken by the patient and it could reflect the surgeon’s preference for opioid prescription more than a patient’s pain trend [8,20]. Because our study was not blinded, patients who received nerve blocks may have been inclined to request fewer opioid pain medications. Conversely, nurses may have been more likely to offer opioids to Group A knowing they did not receive a nerve block, especially in the PACU. This tendency could be attributed to the fact that nerve blocks are promoted to reduce opioid usage during surgery, making them a more attractive option for patients who naturally prefer to avoid opioid drugs. Additionally, our analysis of patients requiring narcotic drug refills post-discharge revealed no statistically significant differences. These findings bolster our hypothesis that the quantity of pills prescribed may be influenced more by the treating physician’s prescription protocol rather than the actual needs of the patients.

Our study has shown that a perioperative nerve block is not clinically beneficial in decreasing postoperative pain during hospitalization. Furthermore, nerve blocks have been found to lead to an increased risk of fall due to muscle weakness [25], along with a possible risk of nerve injury, which can be either neurotoxic, cytotoxic, or traumatic [26]. In a series looking at nerve blocks in pediatric patients, Frawley et al. found that prolonged sensory block might lead to delay in the timely diagnosis and treatment of peripheral nerve injury, in the case of nerve damage following tibial osteotomy for lengthening and tibial torsion correction procedures [27].

For these aforementioned reasons and with a lack of evidence for significant clinical pain relief, peripheral nerve blockade for femoral lengthening should be thoroughly considered on a case-by-case basis. Our study demonstrates non-inferiority of general anesthesia without preoperative nerve blockade during femoral lengthening procedures, in terms of postoperative pain during hospitalization and with equivocal results of its effects after discharge. As such, we can conclude that the choice of applying a nerve block should be discussed between the patient, the surgeon, and the anesthesiologist, while taking into consideration the results of our study.

Our study has several limitations including limitations to its retrospective nature, as all cases were reviewed from patient’s health records. One major limitation is the reliance on pre-registered health records, which may be missing, inaccurate, or inconsistent due to differences in data recording. Moreover, retrospective studies limit the ability for standardized data collection methods. Furthermore, the number of pills prescribed on discharge could be a non-specific method to measure pain upon discharge. Due to no blinding, there may have been changes in practice pattern due to perceived effects of the nerve block from both the perspective of the patient and the treating team. An important consideration in interpreting our results is the choice of the non-inferiority margin set at one standard deviation (SD). While this choice is consistent with common standards in pain research and aimed at capturing clinically relevant differences, selecting a broader margin could potentially overlook subtle yet meaningful differences between treatment groups. Conversely, a narrower margin may detect differences that, although statistically significant, could lack clinical relevance, potentially leading to unnecessary changes in clinical practice. Thus, our chosen margin provides a balanced approach but inherently influences our conclusion of non-inferiority. Another limitation is the lack of randomization inherent in our retrospective study design, which introduces potential biases and may limit the strength of our conclusions. Future studies might explore sensitivity analyses using different margins to further validate these findings.

## Figures and Tables

**Table 1:** Demographic distribution of the cohort.

	Sex			
	Male	Female	Weight	Age
Block (61)	30	31	41.9	20.8
No Block (131)	77	54	41.5	21.6
Total or Average	107	85	41.6	21.4

**Table 2:** Difference in mean pain, maximum pain, total number of pills, and MME in patients receiving femoral nerve block compared to femoral and fascia iliaca nerve block.

	Femoral (33)	Femoral + Fascia Iliaca (28)	*p*
Mean Pain	2	1.4	>0.05
Max Pain	3.3	2.3	>0.05
Total N of Pills	7	13	>0.05
MME	25	19	>0.05

## Data Availability

The raw data supporting the conclusions of this article will be made available by the authors on request.
